# Disengaging the COVID-19 Clutch as a Discerning Eye Over the Inflammatory Circuit During SARS-CoV-2 Infection

**DOI:** 10.1007/s10753-022-01674-5

**Published:** 2022-05-30

**Authors:** Mohammed Moustapha Anwar, Ranjit Sah, Sunil Shrestha, Akihiko Ozaki, Namrata Roy, Zareena Fathah, Alfonso J. Rodriguez-Morales

**Affiliations:** 1 Department of Biotechnology, Institute of Graduate Studies and Research (IGSR), Alexandria University, Alexandria, Egypt; 2 Tribhuvan University Institute of Medicine, Kathmandu, Nepal; 3 Department of Pharmaceutical and Health Service Research, Nepal Health Research and Innovation Foundation, Lalitpur, Nepal; 4 Department of Breast Surgery, Jyoban Hospital of Tokiwa Foundation, Iwaki, Japan; 5 SRM University, SRM Nagar, Kattankulathur, Chengalpattu, Tamil Nadu 603203 India; 6 Kings College London, London, UK; 7 Grupo de Investigación Biomedicina, Faculty of Medicine, Fundacion Universitaria Autonoma de Las Americas, Pereira, Risaralda Colombia; 8 Institución Universitaria Visión de Las Americas, Pereira, Risaralda Colombia; 9 Faculty of Health Sciences, Universidad Cientifica del Sur, Lima, Peru; 10 School of Medicine, Universidad Privada Franz Tamayo (UNIFRANZ), Cochabamba, Bolivia; 11 College of Medicine and Health Sciences, United Arab University, Abu Dhabi, United Arab Emirates; 12 Medical Governance Research Institute, Tokyo, Japan

**Keywords:** COVID-19, SARS-CoV-2, Mitochondria, NOX, TLR, NLRP3, ROS, IL

## Abstract

Severe acute respiratory syndrome coronavirus 2 (SARS-CoV-2) causes the cytokine release syndrome (CRS) and leads to multiorgan dysfunction. Mitochondrial dynamics are fundamental to protect against environmental insults, but they are highly susceptible to viral infections. Defective mitochondria are potential sources of reactive oxygen species (ROS). Infection with SARS-CoV-2 damages mitochondria, alters autophagy, reduces nitric oxide (NO), and increases both nicotinamide adenine dinucleotide phosphate oxidases (NOX) and ROS. Patients with coronavirus disease 2019 (COVID-19) exhibited activated toll-like receptors (TLRs) and the Nucleotide-binding and oligomerization domain (NOD-), leucine-rich repeat (LRR-), pyrin domain-containing protein 3 (NLRP3) inflammasome. The activation of TLRs and NLRP3 by SARS‐CoV‐2 induces interleukin 6 (IL-6), IL-1β, IL-18, and lactate dehydrogenase (LDH). Herein, we outline the inflammatory circuit of COVID-19 and what occurs behind the scene, the interplay of NOX/ROS and their role in hypoxia and thrombosis, and the important role of ROS scavengers to reduce COVID-19-related inflammation.

## INTRODUCTION

**Fig. 1:**
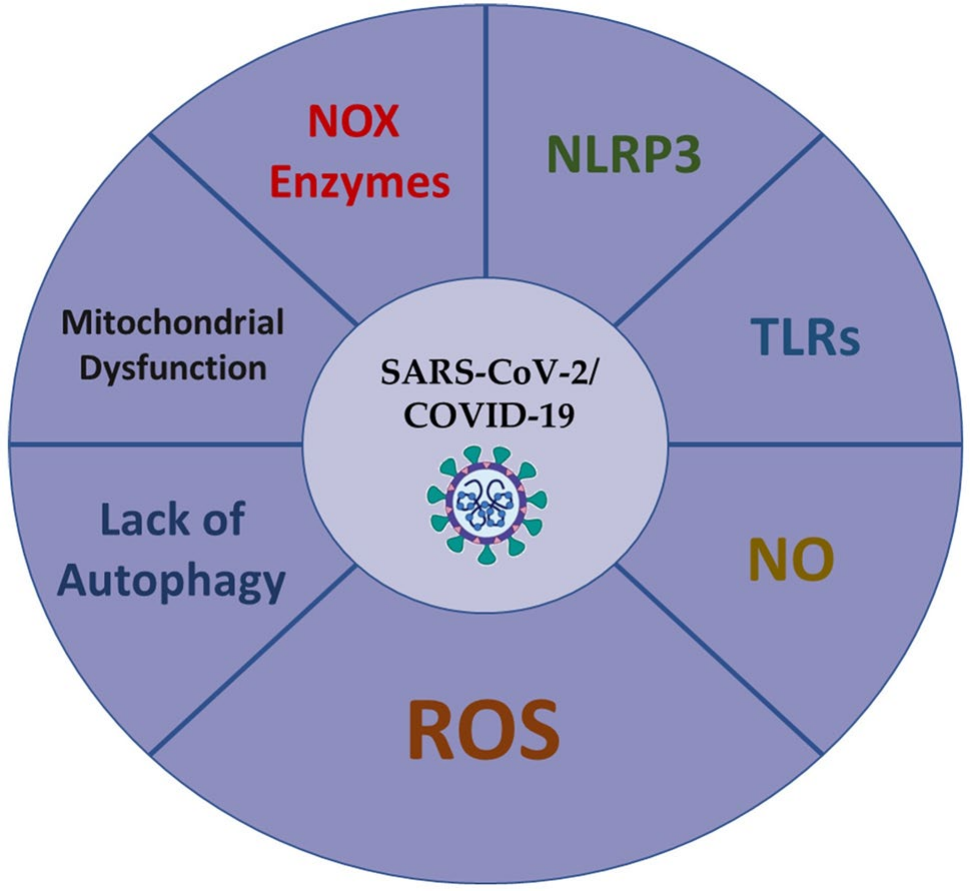
A﻿ summary of the machineries that aggravate COVID-19. **COVID-19**, Coronavirus Disease-19; **NLRP3** = NOD-, LRR- and pyrin domain-containing protein 3; **NO**, Nitric Oxide; **ROS**, Reactive Oxygen Species; **SARS-CoV-2**, Severe Acute Respiratory Syndrome Coronavirus-2; **TLRs**, Toll-Like Receptors.

Coronavirus disease-19 (COVID-19) poses a menace to public health with almost half a billion cases and approximately six million deaths worldwide [1, 2]. Invading the human lungs, severe acute respiratory syndrome coronavirus 2 (SARS-CoV-2) interacts with the mucous membranes across different organs, such as the eyes, nose, and mouth. Older people with comorbidities such as the metabolic syndrome and diabetes experience severe COVID-19 symptoms. Moreover, increased mortality due COVID-19 was attributed to other risk factors such as older age, diabetes, hypertension, and renal disease. For instance, more than 65% of COVID-19 patients had diabetes and cardiovascular diseases, of which 63% were above 60 years [3]. In addition, SARS-CoV-2 damages mitochondria, alters autophagy, reduces nitric oxide (NO), increasing nicotinamide adenine dinucleotide phosphate oxidases (NOX) as well as reactive oxygen species (ROS). In COVID-19, SARS-CoV-2 also activates both toll-like receptors (TLRs) and the NOD^−^, LRR^−^, and pyrin domain-containing protein 3 (NLRP3) inflammasome [4–12]. The SARS-CoV open reading frame 9b (ORF-9b) manipulates the human mitochondrial antiviral signalling molecule (MAVS) to evade the innate host immunity, limit the antiinflammatory response, and overproduce ROS [10, 13]. The NOX protein family produces ROS that enhance viral pathogenicity in inflammatory cells [10, 11]. Mammalian NOX enzymes and subunits include NOX1-5, p22^phox^, p67^phox^, NOXO1 that are elevated in response to angiotensin II (ATII) in the kidneys, heart, and endothelial cells. Such enzymes and subunits are also involved in COVID-19 [14, 15]. Infection with SARS-CoV-2 mediates inflammatory cytokines and chemokines, where ATII-induced interleukin-6 (IL-6) synthesis usually requires NOX-derived ROS [7]. Patients and mice who are NOX2-deficient had enhanced immune response with a tendency to develop autoantibodies with low ROS levels [8, 9]. The activation of TLRs and NLRP3 by SARS‐CoV‐2 induces IL‐6, IL-1β, IL-18, and lactate dehydrogenase (LDH) [4, 5, 16–23]. Currently, research has discussed a higher number of involved systems in COVID-19, but from an individual perspective. Herein, the present review article combines the simultaneous detrimental effects of mitochondrial dysfunction, autophagy, NOX, NO, ROS, NLRP3, and TLRs during COVID-19 (Fig. 1). Moreover, we referred to the potential role of ROS scavengers in COVID-19.

## BEHIND-THE-SCENE IN COVID-19

1. The NOX-Mediated ROS Pathway of Inflammation

**Fig. 2:**
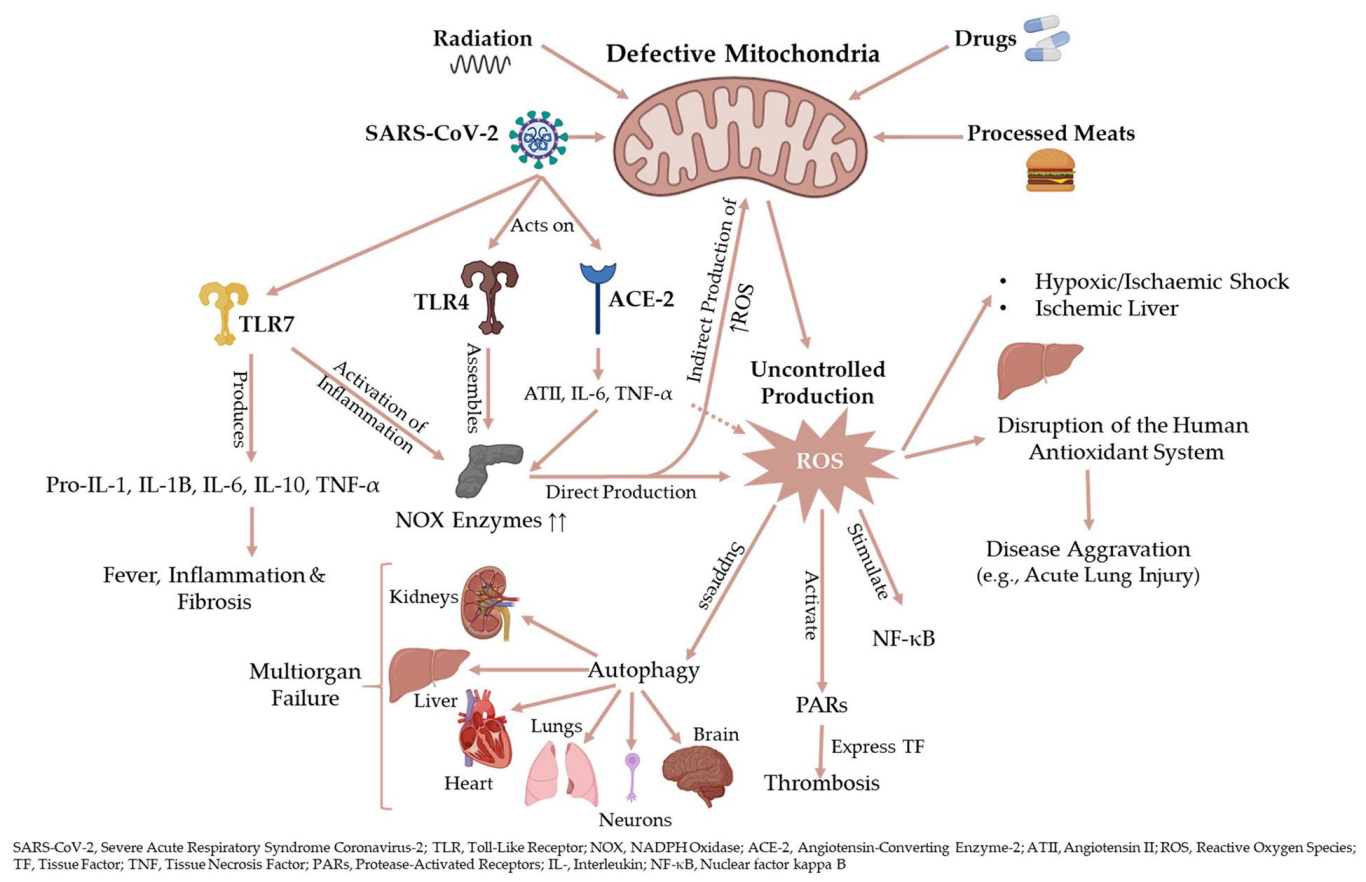
A schematic representation of the interplay between mitochondria and inflammatory related factors with COVID-19 at different levels. **ACE-2**, Angiotensin-Converting Enzyme-2; **ATII**, Angiotensin II; **IL**, Interleukin; **NF-κB**, Nuclear factor kappa B; **NOX**, NADPH Oxidase; **PARs**, Protease-Activated Receptors; **ROS**, Reactive Oxygen Species; **SARS-CoV-2**, Severe Acute Respiratory Syndrome Coronavirus-2; **TF**, Tissue Factor; **TLR**, Toll-Like Receptor; **TNF-ɑ**, Tissue Necrosis Factor-Alpha.

**Fig. 3:**
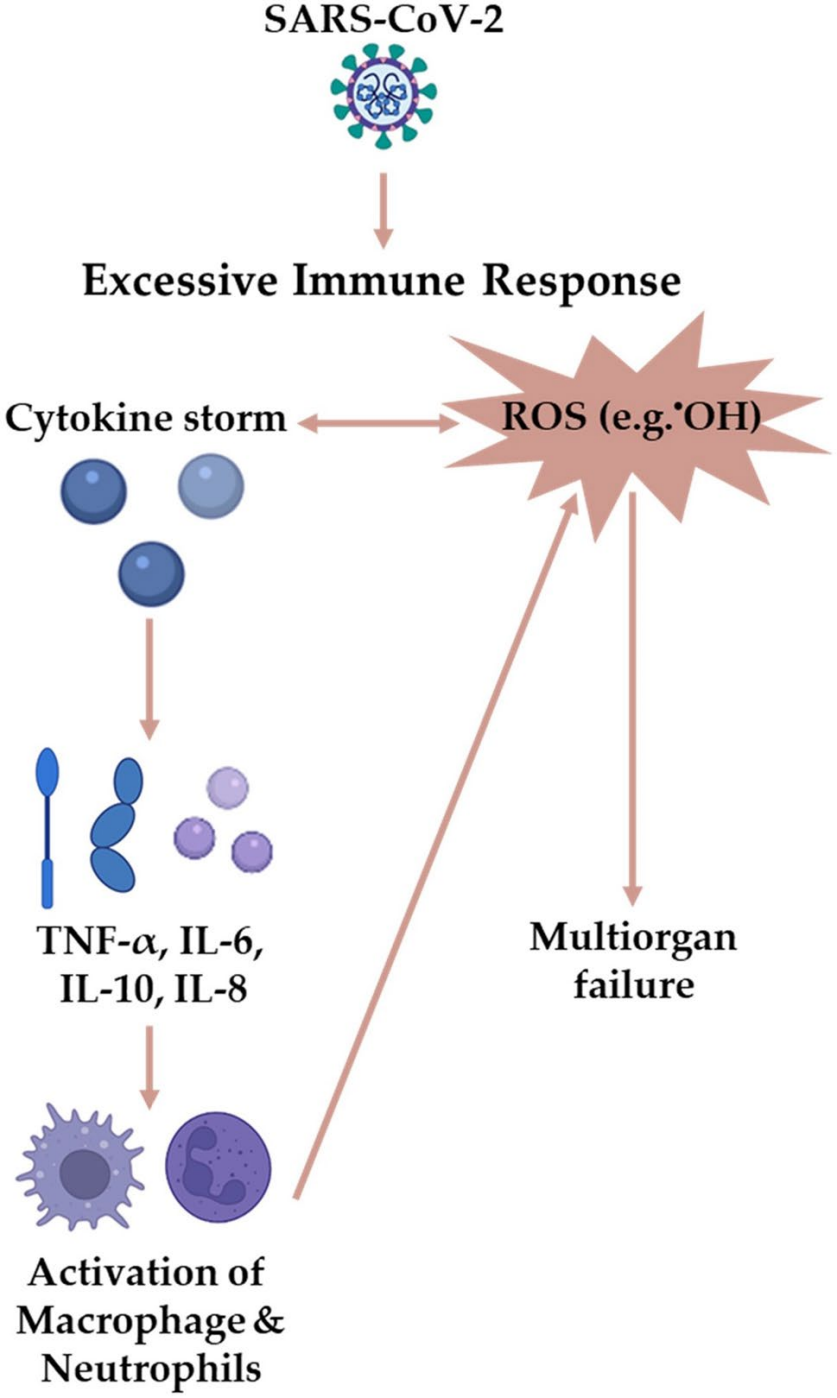
^•^OH as the potent ROS family member that lead to multiorgan failure in COVID-19. **IL**, Interleukin; **ROS**, Reactive Oxygen Species; **SARS-CoV-2**, Severe Acute Respiratory Syndrome Coronavirus-2; **TNF-ɑ**, Tissue Necrosis Factor-Alpha.

The dysregulation of NOX signalling is evident in COVID-19 patients with comorbidities, including obesity, diabetes, coronary artery disease, and heart failure [24]. In COVID-19 patients with acute respiratory distress syndrome (ARDS), ATII increases NOX and causes vasoconstriction and thrombosis *via* ROS, IL-6, tumour necrosis factor-Alpha (TNF-α), and other cytokines (Fig. 2) [25, 26]. The generation of NOX-dependent ROS elevates TNF-α, transforming growth factor-beta 1 (TGF-β1), ATII, and plasminogen activator inhibitor-1 (PAI-1), all of which are increased in COVID-19 patients [24, 27–30]. Numerous endogenous and exogenous processes produce ROS, such as NOX, the electron transport chain, xanthine oxidase, smoking, heavy metals, drugs, processed meat, and radiation (Fig. 2) [31]. Interferon-Gamma (IFN-γ) and ATII in vascular smooth muscle trigger NOX1 expression, while hypoxia/ischaemia and TNF-α stimulate NOX4 [32, 33]. Endosomal NOX2 produces the proinflammatory leukotriene B4 (LTB4) and increases the levels of IL-6 and ROS in virus-mediated pathogenicity [10, 34–38]. For example, influenzae A virus causes significantly less lung injury in the absence of NOX2, highlighting that NOX2-mediated ROS stimulates viral infection [35, 39]. In COVID-19, SARS-CoV-2 upregulates both ACE and ATII and therefore activates the phagocytes, metabolises haemoglobin, and causes hyperferritinaemia to produce hydroxyl radical (^•^OH), increasing the likelihood of inflammation and thrombosis (Fig. 3) [40–51].

The formation of ^•^OH correlates with oxidative stress products such as 4-hydroxynonenal and malondialdehyde guanine adducts of DNA, which also are the products of the radical oxidation of phospholipids, related to COVID-19 dyslipidaemia [52–55]. Reactive oxygen species interact with lipids, carbohydrates, proteins, and nucleic acids, causing permanent destruction or alterations in their functions [56]. Hydroxyl radical is the most reactive and most toxic ROS that causes severe cellular damage by strongly interacting with DNA, carbohydrates, proteins, and lipids [57–60]. Haemochromatosis in different diseases (e.g., ageing and Parkinson’s disease) has gained attention because iron catalyses the formation of ^•^OH [61–64]. Hydroxyl radical directly reacts with all DNA components, such as purine and pyrimidine bases, deoxyribose sugar backbone and causes single and double stranded breaks in DNA strand breaks and chemical modifications of nucleobases or nucleotides [60, 65, 66]. The uncontrolled production of ROS significantly contributes to infectious, inflammatory, and numerous chronic disorders. This evidence underpins the current hypothesis that NOX is an essential regulator in COVID-19 pathogenesis, and that blocking the expression of NOX might hinder the production of ATII-induced ROS and IL-6, minimising inflammation and tissue injury (Fig. 2).

2. The Inflammatory Role of NLRP3

The tissues of postmortem COVID-19 patients show the active NLRP3 inflammasome and its products, including IL-1β, IL-18, and LDH [16–23]. Acute and chronic respiratory diseases, traumatic brain injury, acute kidney injury (AKI), and chronic kidney disease (CKD) also reported the involvement of the NLRP3 inflammasome [67]. Viral infections, metabolic abnormalities, tissue damage, and dysfunctional mitochondria generate ROS (e.g., ^•^OH) that activate the NLRP3 inflammasome, triggering the production of proinflammatory cytokines [68–73]. Fortunately, mitochondria-targeted antioxidants such as molecular hydrogen (H_2_) can suppress the production of mitochondrial ^•^OH, and therefore inhibit the expression of NLRP3 inflammasome, caspase-1, and IL-1β [74]. Molecular hydrogen is a potent scavenger that selectively scavenges ^•^OH without adverse effects on the human body [75]. A recent multicentre trial revealed that the inhalation of hydrogen–oxygen gas mixture reduced COVID-19-related acute and chronic inflammation [76]. The intraperitoneal H_2_-rich saline suppressed the activation of the NLRP3 inflammasome, the activity of nuclear factor kappa-light-chain-enhancer of activated B cells (NF-*κ*B), and the production of TNF-α and IL-1β in a mouse model with acute pancreatitis. Moreover, H_2_-rich saline improved the survival rate and ameliorated intestinal damage and inflammatory response, oedema, and apoptosis ameliorated intestinal ischaemia/reperfusion-mediated coagulopathy in rats. Molecular hydrogen-rich saline inhibited the activation of NF-*κ*B and NLRP3 inflammasomes in peripheral blood mononuclear cells (PBMCs) [77]. Given this, H_2_ may reduce the SARS-CoV-2-induced inflammation by inhibiting the NLRP3 cascade and the release of proinflammatory cytokines.

3. The Nitric Oxide (NO)/ROS Imbalance

**Fig. 4:**
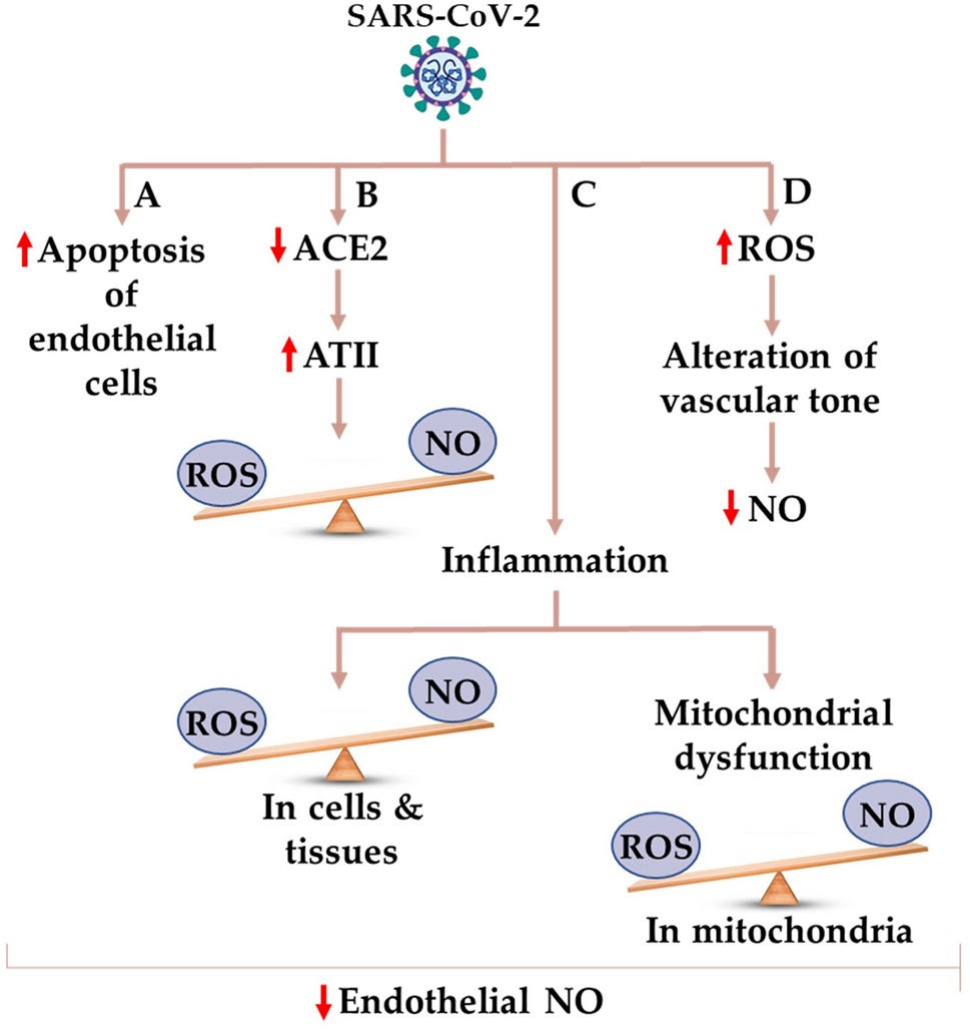
The potential role of ROS/NO imbalance in reducing endothelial NO during COVID-19. **ACE-2**, Angiotensin-Converting Enzyme-2; **ATII**, Angiotensin II; **NO**, Nitric Oxide; **ROS**, Reactive Oxygen Species; **SARS-CoV-2**, Severe Acute Respiratory Syndrome Coronavirus-2.

Persistent inflammation due to COVID-19 disturbs the nitric oxide (NO)/ROS balance and causes multiorgan failure [78]. Patients with COVID-19 and common comorbidities (e.g., hypertension and diabetes) displayed significantly reduced endothelial NO, suggesting a strong relationship with acute lung injury (ALI) and NO/ROS imbalance [79–85]. Severe acute respiratory syndrome coronavirus 2 downregulates the expression of angiotensin-converting enzyme 2 (ACE2), producing proinflammatory cytokines and ROS that cause excessive inflammatory responses and lower the levels of NO by causing endothelial cell apoptosis (Fig. 4) [86–89]. Viral SARS-CoV-2 particles easily bind their protein spikes and enter into the cells due to the higher expression of ACE-2 receptors. Hence, people with impaired metabolic health are more prone to COVID-19 and comorbidities [3]. Severely ill COVID-19 patients exhibit excessive mitochondrial ROS that lead to mitochondrial dysfunction, reducing the production and bioavailability of NO by the activation of nuclear factor kappa-light-chain-enhancer of activated B cells (NF-_k_B), AP-1 as well as the overexpression of cytokines and adhesion molecules (Fig. 2) [90–92]. The NO donor S-nitroso-N-acetylpenicillamine (SNAP) significantly inhibited cysteine proteases encoded by SARS-CoV-1 ORF1a and the membrane fusion of offspring virus S protein, decreasing viral replication by > 80% in VeroE6 cells [93–97]. Both SARS-CoV-2 and SARS-CoV exhibit a high degree of similarity in the receptor-binding domains of the spike proteins [98, 99]. Consequently, inhaled NO may prevent SARS-CoV-2 infection or treat mild, moderate, or severe COVID-19 patients, and could be used as an adjuvant therapy in mechanically ventilated patients (Fig. 4) [83, 100, 101].

4. Mitochondrial Dysfunction and Autophagy

Hypoxia and other inflammatory mediators impair the function of mitochondria during COVID-19 [102, 103]. Mitochondrial dysfunction is a potential source of ROS that affect healthy mitochondria and promote cell death [104]. Mitochondria have emerged as critical dynamic organelles to maintain cellular homeostasis, metabolism, innate immune response, and determine the severity of viral infections [105]. Mitochondrial dynamics such as fusion, fission, and mitophagy protect against environmental insults; although, they are susceptible to viral infections, due to viral proteins or physiological alterations (e.g., disruption of Ca^2+^ homeostasis, endoplasmic reticulum stress, oxidative stress, and hypoxia) [106–108]. By interfering with mitochondria, viruses distort mitochondrial functions to create a favorable stressful environment for viral proliferation (i.e., low and higher amounts of mitochondrial ATP and ROS, respectively) and impeding mitochondria-associated antiviral signaling [109]. Defective mitochondria are a potential source of ROS that can also lead to damage of healthy mitochondria. Therefore, disturbances of the rapid clearance of dysfunctional mitochondria create higher levels of ROS, promoting cell death [102, 104, 110, 111]. Afterwards, viruses (e.g., SARS-CoV-2) start to proliferate and propagate *via* changing potential targets, including NLRP3 inflammasome and autophagy [112].

In COVID-19-related sepsis, the SARS-CoV-2-host interaction releases the cytokine storm that ultimately leads to multiorgan failure [113]. The proinflammatory cytokine TNF-α increased mitochondrial ROS mediated by mitochondrial damage in human umbilical vein endothelial cells (HUVECs) [114]. Similarly, COVID-19 significantly upregulates TNF-α alongside other cytokines and chemokines (Figs. 1 and 3). Accordingly, SARS-CoV-2 presumably counteracts the antiviral response by upregulating TNF-α and causing mitochondrial ultrastructural abnormalities to produce higher amounts of ROS [115]. Viruses modulate mitochondria-mediated antiviral immune responses by altering autophagy, mitophagy, and cellular metabolism to facilitate their proliferation [112].

Autophagy is an essential target in SARS-COV-2-mediated COVID-19 [112]. The possible inhibition of autophagy by SARS-CoV might elaborate more the pathophysiological role of mitochondrial dysfunction during COVID-19. Cells adopt autophagy (i.e., a self-destruction mechanism) to remove dysfunctional and superfluous cellular components *via* the initiation and elongation of isolation membrane, autophagosomes formation, and fusion and degradation of autophagosome-lysosome [112]. Mitochondria regulate autophagy to remove harmful components by producing ROS, whereas autophagy controls mitochondrial homeostasis using mitophagy [116, 117]. The lack of normal autophagy due to viral infections leads to mitochondrial dysfunction and ROS generation (Fig. 2) [118]. Cardiovascular, neurodegenerative, chronic liver, and kidney diseases also confirmed the interaction between autophagy deterioration, mitochondrial dysfunction, and ROS generation [119–122]. These data support the fact that loss of normal autophagy might be one of the primary contributors to SARS-CoV-2 infection in disturbing the mitochondrial homeostasis. However, numerous studies reported that SARS-CoV, SARS-CoV-2, Middle East respiratory syndrome coronavirus (MERS-CoV), and mouse hepatitis virus (MHV) induce and inhibit autophagy. Further research on modulating autophagy (i.e., induction or inhibition of autophagy) would elaborate the consequences on SARS-CoV-2 treatment [123–132].

5. Loss of Autophagy and ROS

Elderly COVID-19 patients exhibit a vulnerable antioxidant defence and an exaggerated oxidative damage. The onset of ARDS in COVID-19 patients requires the activation of the “ROS machinery” combined with innate immunity to facilitate NF-κB, exacerbating the proinflammatory host response (Fig. 2) [133]. The overproduction of ROS significantly disturbed the antioxidant system during the SARS-CoV pathogenesis, severity, and progression of the respiratory disease *in vitro* and *in vivo* [134, 135]. Humans share age-related loss of autophagy or shocking exposure to ROS. Autophagy may contribute to the ageing phenotype, denoting that ageing alters the adaptive immune response and the proinflammatory state of the host [136]. For example, older mice severely experienced SARS-CoV-induced lung lesions than younger mice [137]. Older macaques upregulate virus-host response with inflammation due to differential gene expression with NF-_k_B as a central player [137]. Elderly patients also had significantly higher incidence of multilobe lesions than young and middle-aged COVID-19 patients [138]. The concurrent decline in mitochondrial dysfunction due to the inhibition of autophagy and the predisposing comorbidities in elderly patients, might explain why old COVID-19 patients show severe clinical manifestations that eventually lead to multiorgan failure compared to younger patients (Fig. 2). The World Health Organization declared that currently approved medications (e.g., clozapine, glyburide, carbetapentane) could be used for the treatment of COVID-19, by targeting the NLRP3 inflammasome and autophagy to inhibit the propagation of SARS-CoV-2 [139–143].

6. The Possible Crosstalk Between TLRs, NOX, and ROS

Evidence supports the association between NOX, ROS, inflammatory mediators, and SARS-CoV-2 pathogenesis as well as the relationship between ROS signalling with TLR4 activation during TLR4/NOX interaction (Fig. 2) [144, 145]. The administration of diphenyleneiodonium chloride (DPI) suppressed the upregulation of TLR2, 4, and 9 in alcohol-induced fatty liver injury [146]. Human cells highlighted the potential role of NOX2 inhibitors in viral infections. In respiratory syncytial virus, rhinovirus, and human immunodeficiency virus (HIV), TLR7 activates NOX2 to produce ROS and modifies the single cysteine residue of TLR7, inhibiting the key antiviral and humoral signalling [147]. The syncytial viral cytoplasmic components recognise TLR7 and other sensor molecules; the mitochondria produce large amounts of **·**OH that oxidise mitochondrial DNA, driving the cascade from NLRP3 to the release of proinflammatory cytokines (Fig. 2) [72, 148].

Severe acute respiratory syndrome coronavirus 2 binds to TLRs to activate and regulate pro-IL-1, NLRP3, IL-1β, IL-6, IL-10, and TNF-α. Such cascade causes lung inflammation and fibrosis, suggesting that the TLR pathways are protective mechanisms in SARS-CoV infections [149–151]. Toll-like receptors (e.g., TLR3, 4, 7, 8, and 9) identify many viral conserved patterns where myeloid differentiation primary response 88 (MyD88)—an essential component of the TLR pathway—assembles NOX to generate ROS in neutrophils and macrophages (Fig. 2) [152, 153]. Myeloid differentiation primary response 88 activates the TIR-domain-containing adapter-inducing interferon (TRIF)‐dependent signalling to activate the IFN-1, NF‐_k_B, and mitogen-activated protein kinase (MAPK) pathway [154]. The activation of the TLR-MyD88 downstream signalling and NF-_k_B is a hallmark of SARS-CoV infections, where the inhibition of NF-_k_B significantly reduced respiratory coronavirus infection and increased survival in mice [151, 155].

Convalescent SARS-CoV-infected patients experienced mitochondrial- and ROS-responding gene upregulation [144]. For example, ROS/NF‐_k_B/TLR (mainly TL4) signalling pathways lead to ALI upon triggering by SARS-CoV. The TLR4-TRIF-TRAF6 pathogenic pathway mediates the severity of ALI. The loss of TLR4 or TRIF expression protected mice from H_5_N_1_-induced ALI, indicating that the severity of ALI depends on ROS and innate immunity.

## THE POTENTIAL ROLE OF THE NOX/ROS INTERPLAY IN MEDIATING HYPOXIA, ISCHAEMIC INJURY, THROMBOSIS, AND FIBROSIS IN COVID-19

Severe hypoxia occurring during the COVID-19 cytokine storm is the leading cause of myocardial and liver damage, toxic encephalopathy, extremity ischaemia, and abnormal coagulation [156–159]. Although the activation of NOX in pulmonary endothelium mediates an increase in ischaemia-mediated ROS, data remain scarce to support the role of the NOX family in hypoxia/ischaemia in COVID-19 patients [160, 161]. A murine model of coronary artery ligation showed that NOX2 led to adverse cardiac injury [162]. Rhinovirus, SARS-CoV, and the anoxia of human platelets generate NOX2-dependent ROS *in vitro* [163]. The genetic deletion of NOX2 quenched the cognitive deficits promoted by intermittent hypoxia and oxidative stress in mice [164, 165]. Mice transplanted with p47^phox^-deficient bone marrow had decreased levels of lung ischaemia and proinflammatory cytokines [166]. Apocynin—NOX2 inhibitor—reduced vascular permeability in sheep, and aborted ischaemic lung and hepatic injury, cell necrosis and tissue injury, cytokine release, and ROS production in different murine models [167–173]. These data highlight that the inhibition of NOX, ROS, or p47^phox^ could hold promise for designing effective molecules to limit the ischaemic injury in COVID-19 patients [7, 103, 174].

1. Brain Ischaemia

Patients with COVID-19 present with ischaemic strokes. Brain ischaemic stroke comprises more than 80% of all strokes and occurs due to an immediate halting of blood flow by middle cerebral artery blockade [175, 176]. The excessive production of ROS aggravates oxidative stress and contributes to brain damage during ischaemia, suggesting that decreasing ROS might be helpful in the management of cerebral stroke (Fig. 1) [177–180]. Studies demonstrated that NOX1, NOX2, NOX4, and NOX5 are associated with cerebral disorders and ROS release [181–186]. The genetic deletion of NOX2 had protective effects against cerebral stroke in middle cerebral artery occlusion (MCAO) model. Functional NOX2-deficient and NOX2 knockout (KO) mice had significant reduction of oedema, lesion volume, and blood–brain barrier (BBB) leakage, postischaemic inflammatory gene expression and oxidative stress markers, and better neurological function during cerebral ischaemia [187–190]. Mouse model of retinal ischaemia with NOX2-deficient hippocampal neurons experiences low ROS levels upon exposure to oxygen/glucose deprivation (OGD) with attenuated neuronal cell death [191]. Consequently, the treatment of stroke should adopt an effective NOX inhibitory strategy, especially NOX2. However, extensive research that simulates the human biological system is crucial to validate the data emerging from *in vivo* models given the small organs and the relatively large penumbra in the lesioned tissues.

2. Thrombosis and Fibrosis

Microthrombosis, pulmonary embolism, endothelial failure, and disseminated intravascular coagulation (DIC) are reported in COVID-19 patients [7, 192–196]. Viruses activate the coagulation pathway to overproduce proinflammatory cytokines *via* proteinase-activated receptors (PAR1 and PAR2) mediated by mitochondrial ROS [196–201]. Both PAR1/PAR2—expressed on platelets, endothelial and epithelial cells, and vascular and nonvascular smooth muscles—are involved in inflammation [202–205]. The upregulation of the NOX subunit p22^phox^ in endothelial cells generates ROS that promote PAR1- and PAR2-mediated tissue factor (TF) induction, causing acute and chronic inflammation (Fig. 2) [206–209]. During inflammation, iron (III) generate ^•^OH that convert soluble plasma fibrinogen into abnormal fibrin clots in the form of dense matted deposits resistant to enzymatic degradation (i.e., blood coagulation) (Fig. 4) [210–212]. Tissue-plasminogen activator (tPA) downregulates both IL-1α and IL-1β in endothelial cells during inflammation [213, 214]. Three mechanically-ventilated COVID-19 patients demonstrated that tPA has a therapeutic role in ARDS, showing a transient improvement in the ratio of arterial oxygen partial pressure/fractional inspired oxygen [215]. However, this improvement is lost after the end of treatment due to the fact that NOX-dependent ROS inhibits tPA activity, leading to thrombosis [216, 217].

The biopsies of liver and lung injury in deceased COVID-19 patients showed severe inflammatory responses with higher levels of IL-2, IL-6, IL-8. IL-10, and IFN-γ [218]. Profibrotic responses are triggered upon the activation of PAR1 and PAR2, inducing the release of NF-_k_B and IL-6, IL-8, and MCP-1 that contribute to leucocyte recruitment during SARS-CoV-2 infection as well (Fig. 1) [219]. The direct upregulation of PAR (i.e., PAR2) in chronic liver disease and pulmonary fibrosis increases the production of ROS, enhancing fibrogenesis by inducing hepatocyte apoptosis, airway obstruction, and lung oedema [209, 220–223]. This is consistent with the fact that PAR-2-deficient mice showed reduced inflammation and improved survival [224, 225]. Therefore, it is expected that PAR-2-dependent ROS could contribute to lung and liver injuries in COVID-19 patients, especially with predisposing diseases such as liver disease, leading to immunosuppression and disease aggression [25, 226, 227].

## COULD ROS SCAVENGERS BE EFFECTIVE AGAINST COVID-19?

Natural compounds such as lycopene, polyphenols, quercetin, phloretin, berberine, and sulforaphane show a preventive potential against SARS-CoV-2 infection [228–231]. The lecithinised superoxide dismutase (PC-SOD) enzyme possesses excellent bioavailability, safety (confirmed in phase I and II studies), and modulatory effect to reduce the harms of oxidative stress in COVID-19 [232–236]. It is a synthetic product with long-life and high bioavailability compared to non-lecithinsed forms of the enzyme [237, 238]. For example, the intravenous administration of PC-SOD was safe and suppressed pulmonary emphysema and fibrosis, lung inflammation or ARDS, and activation of proteases, and the expression *in vitro* and in animal models [234, 239–243]. The lecithinised superoxide dismutase reduced serum LDH and surfactant protein A in patients with stage III-IV idiopathic pulmonary fibrosis without significant side effects. It would exert a more pulmonary protective effect if administered earlier during the course of the disease [233].

## CONCLUSIONS AND FUTURE DIRECTIONS

This review has shed light on the close relationship between mitochondrial dysfunction, NOX, ROS, NLRP3 inflammasome, TLRs, and NO as the “inflammatory circuit” of COVID-19. The lack of normal autophagy leads to central problems such as mitochondrial dysfunction and the production of ROS. Subsequently, there could be an interplay between autophagy and SARS-CoV-2, but the exact nature of such an interaction remains unclear.

The proposed crosstalk between ROS and NOX during SARS-CoV-2 infection unequivocally constitutes an emerging molecular analysis and drug design route for COVID-19. Other probable interfering signalling pathways (i.e., PAR, TLR-MyD88, ROS/NF‐_k_B/TLR, and TLR4/TRIF/TRAF6) take place during SARS-CoV-2 pathogenesis. The coronavirus proteases, especially 3C-like protease (Mpro or 3CLpro), are attractive antiviral drug targets because they are essential for coronaviral replication. Such antiviral drugs would inhibit viral replication and the dysregulation of signalling cascades in infected cells that may lead to the death of healthy cells [6].

Future investigations may unveil the mitochondrial innate antiviral signalling during COVID-19, SARS-CoV-2–host interactions, and how SARS-CoV-2 exploits alterations to the mitochondrial morphophysiology to its benefit [244, 245]. The inhibitors of NOS and ROX might be promising compounds to reduce the SARS-CoV-2-related hyperinflammatory states during the cytokine response, vascular hyperpermeability, microthrombosis, tissue injury/ischaemia and fibrosis, and multiorgan failure. Nevertheless, the essential functions of NOX and ROS in normal physiology should be considered. The use of antioxidants may face potential challenges, such as physiological interferences, biological functions of NOX/ROS, lack of target access, and the inability to attain adequate ROS concentrations.

## Data Availability

Not applicable.

## Abbreviations

ACE2: Angiotensin-converting enzyme 2
ALI: Acute lung injury
ARDS: Acute respiratory distress syndrome
ATII: Angiotensin II
BBB: Blood-brain barrier
CGD: Chronic granulomatous disease
COVID-19: Coronavirus disease-19
DIC: Disseminated intravascular coagulation
DPI: Diphenyleneiodonium chloride
HIV: Human immunodeficiency virus
HUVECs: Human umbilical vein endothelial cells
IFN-I: Type I interferon
IFN-α: Interferon-alpha
IFN-γ: Interferon-gamma
IL-6: Interleukin-6
KO: Knockout
LTB4: Leukotriene B4
MAPK: Mitogen-activated protein kinase
MAVS: Mitochondrial antiviral signalling molecule
MCAO: Middle cerebral artery occlusion
MI: Myocardial infarction
MyD88: Myeloid differentiation primary response 88
NF-_κ_B: Nuclear factor kappa-light-chain-enhancer of activated B cells
NO: Nitric oxide
NOX: Nicotinamide adenine dinucleotide phosphate oxidases
OGD: Oxygen/glucose deprivation
ORF-9b: Open reading frame 9b
PAI-1: Plasminogen activator inhibitor-1
PAR1: Proteinase-activated receptor 1
PAR2: Proteinase-activated receptor 2
PKC: Protein kinase C
ROS: Reactive oxygen species
SARS-CoV-2: Severe acute respiratory syndrome coronavirus 2
TF: Tissue factor
TGF-β1: Transforming growth factor-beta 1
TLRs: Toll-like receptors
TNF-α: Tumour necrosis factor-alpha
tPA: Tissue-plasminogen activator
TRIF: TIR-Domain-containing adapter-inducing interferon
